# An ethnographic study of sociocultural determinants and health system gaps in chronic venous disease in Calabria

**DOI:** 10.1038/s41598-025-15771-6

**Published:** 2025-08-12

**Authors:** Davide Costa, Raffaele Serra

**Affiliations:** 1 Department of Medical and Surgical Sciences, Magna Graecia University of Catanzaro, 88100 Catanzaro, Italy; 2 Interuniversity Center of Phlebolymphology (CIFL), “Magna Graecia” University, 88100 Catanzaro, Italy

**Keywords:** Chronic venous disease, Ethnography, Medical anthropology, Health system, Sociocultural determinants, Calabria, Italy, Diseases, Health care

## Abstract

Chronic Venous Disease (CVD) is a highly prevalent condition often explored through a biomedical lens, emphasizing pathophysiology and clinical management. However, less attention has been paid to the sociocultural dimensions that shape its perception, experience, and treatment. This ethnographic study investigates how social, cultural, and structural factors influence CVD outcomes in Calabria, a historically underserved region in Southern Italy. Through participant observation and in-depth interviews with patients, caregivers, and community health workers, this paper reveals patterns of delayed diagnosis, stigma, and therapeutic non-compliance tied to cultural beliefs, gender dynamics, and systemic healthcare gaps. Findings suggest the need for culturally embedded interventions and health policies that recognize the lived realities of CVD patients. This study contributes to the emerging intersection of anthropology and preventive medicine, reinforcing the importance of viewing chronic diseases within their broader sociocultural contexts.

## Introduction

Chronic Venous Disease (CVD) is a widespread and progressive vascular condition that significantly impacts quality of life (QoL). Its severity ranges from cosmetic issues to serious complications like venous ulcers. Prevalence varies globally due to differing methods and definitions, with varicose veins affecting up to 57% of men and 77% of women^1–3^. CVD is marked by venous hypertension and impaired blood flow in the lower limbs, leading to symptoms such as heaviness, pain, swelling, and cramps, which can occur even without visible signs. These symptoms often worsen with prolonged standing or sitting and are sometimes relieved by elevation or compression^1–4^.

The burden of CVD extends far beyond physical discomfort. Studies have shown that the chronic pain, restricted mobility, and cosmetic concerns associated with the disease lead to decreased physical activity, poor sleep, and psychosocial distress. Patients frequently report limitations in daily activities, reduced work productivity, and social withdrawal^5^. While considerable research has addressed CVD clinical features, few studies interrogate the sociocultural and structural factors influencing disease management and health-seeking behavior.

Calabria, located in Southern Italy, provides a compelling case study due to its socioeconomic disparities, aging population, and longstanding structural challenges in healthcare access^5^. The region is marked by pronounced economic and social disparities, high unemployment rates, and significant outmigration—especially among the youth. It has one of the oldest populations in Italy and suffers from longstanding healthcare system challenges, including provider shortages, geographic inaccessibility, bureaucratic inefficiencies, and uneven distribution of specialist care. Health services in rural and semi-urban areas are often fragmented, and structural obstacles—such as lack of transportation and long waiting lists—contribute to chronic underutilization of care. These systemic issues contribute to regional health inequities and foster an environment in which chronic illnesses like CVD may go untreated or be mismanaged for extended periods. Calabria’s marginalization within the broader Italian health system also makes it an important site for investigating how structural violence and social determinants of health shape disease trajectories. Calabria’s marginalization within Italy’s health system makes it a key case for studying how structural violence and social determinants shape illness. Widespread poverty, youth outmigration, and poor healthcare rankings create major challenges for managing chronic diseases. The region suffers from underfunded health infrastructure, staff shortages, and high rates of untreated illness, forcing many to seek care elsewhere. These issues are worsened by limited telehealth access, particularly among the rural elderly^6,7^.

These systemic pressures intersect with Calabria’s strong herbal and faith-based healing traditions. Local ethnobotanical practices remain widely used alongside folk healing rituals—cultural universes that shape how people perceive and manage illness, especially in areas with limited formal service access.

Medical anthropology and ethnography provide critical tools for analyzing how illness is experienced, narrated, and shaped by broader social, political, and economic forces^8^. Foundational scholars such as Paul Farmer^9,10^ have emphasized the importance of understanding disease not only as a biological event but also as a socially produced phenomenon deeply entangled with inequality, history, and power relations. Ethnographic inquiry allows for the exploration of patient narratives, not simply as individual stories, but as situated accounts that reflect structural violence, health system failures, and local meaning-making processes. Scholars like Arthur Kleinman^11^ have shown that attention to illness experience can reveal how people navigate suffering, stigma, and care, often in settings marked by medical pluralism and systemic neglect. Furthermore, João Biehl’s^12^ work on therapeutic itineraries and Veena Das’s^13^ research on the “eventfulness” of illness in everyday life underscore how marginalization and bureaucratic violence can shape access to care, perceptions of the body, and experiences of chronicity. In this light, examining how people in Calabria interpret and respond to Chronic Venous Disease offers a lens into the entwinement of corporeal distress, cultural idioms, and institutional shortcomings^14^.

Based on these considerations, this research is guided by the following questions: How do social, cultural, and structural factors influence the perception, experience, and treatment of Chronic Venous Disease in Calabria? What do these influences reveal about the limitations of a predominantly biomedical approach to chronic illness? By situating CVD within its lived and systemic contexts, this study contributes to both vascular medicine and the anthropology of health by highlighting how care is shaped not only by diagnosis and treatment but also by social location, belief systems, and everyday constraints.

## Materials and methods

### Study design and setting

This study employed a qualitative ethnographic design, conducted between October 2018 and September 2024 in two distinct geographic and sociocultural contexts within the Calabria region of Southern Italy: (1) an urban neighborhood in the city of Catanzaro, and (2) a rural village in the Sila mountain range. These sites were selected to capture a diverse range of experiences across varying levels of healthcare access and infrastructural development. Calabria is characterized by significant socioeconomic disparities and limited health system resources, making it a relevant case for examining structural determinants of chronic illness.

The extended duration (6 years) of fieldwork reflects a multi-phased ethnographic approach involving periods of active immersion interspersed with intervals of follow-up, reflection, and iterative engagement. This prolonged timeline allowed the researcher to build trust and rapport, particularly with older adults in underserved areas, and to revisit participants over time to trace evolving illness narratives and healthcare trajectories.

This study forms part of a larger interdisciplinary research initiative examining chronic illness experiences in Southern Italy. While that broader project includes multiple chronic conditions, this manuscript focuses specifically on the sociocultural dimensions of CVD, providing a detailed case study within the broader fieldwork.

### Participant recruitment and sampling strategy

Participants were recruited using purposive sampling, with the aim of capturing a wide range of illness experiences and sociocultural perspectives on Chronic Venous Disease (CVD). Eligibility criteria included adults aged 50 years or older with a clinical diagnosis of CVD (CEAP classification ≥ C2), and either currently receiving treatment or having a history of symptoms associated with the condition. Recruitment was facilitated through local health clinics, community health workers, and snowball sampling.

Rapport was developed through repeated, informal interactions in both clinical and community settings, with trust reinforced by the ethnographer’s consistent presence, use of local dialects, and attention to participants’ social and cultural contexts.

A total of 27 individuals participated in the study:

- 18 patients diagnosed with CVD (age range: 50–85; 11 women, 7 men).
- 4 family caregivers.
- 5 community health workers (CHWs) engaged in informal or semi-formal care roles within their communities.

Verbal informed consent audio-recorded was obtained from all participants prior to participation. Pseudonyms are used in all fieldnotes and transcripts to ensure anonymity.

## Data collection procedures

Fieldwork was conducted primarily by D.C., who served as the lead ethnographer and was responsible for participant observation, interviewing, fieldnote documentation. R.S. was also involved in biomedical assessment.

Data were gathered through three primary methods:

1. Participant observation.

Fieldwork involved extended participant observation in public outpatient clinics, community health centers, and private domestic settings. Observations focused on care routines, interpersonal dynamics, healthcare interactions, and embodied experiences of illness. Detailed fieldnotes were recorded immediately after each observation session, capturing both verbal exchanges and non-verbal communication (e.g., body language, spatial positioning, care gestures).

2. In-depth interviews.

Semi-structured, in-depth interviews were conducted with all participants, using guides developed according to grounded theory principles. While the overarching framework explored the sociocultural, behavioral, and structural dimensions of chronic illness, the themes were adapted to suit the specific roles and experiences of each group.

For patients diagnosed with Chronic Venous Disease (*n* = 18), interviews explored how individuals interpreted and narrated their symptoms, their experiences with healthcare-seeking behavior and treatment adherence, and the emotional and social effects of visible symptoms. Participants also discussed their use of traditional or alternative therapies, their perceptions of the healthcare system and its actors, and the influence of gender norms on how illness was embodied and expressed.

Interviews with family caregivers (*n* = 4) centered on their daily caregiving routines and the responsibilities they assumed, particularly in relation to navigating medical appointments and home care. These conversations also addressed the emotional toll of caregiving, intergenerational tensions around health decisions, and the need to mediate between elders’ traditional beliefs and biomedical recommendations. Caregivers frequently commented on the availability—or lack—of institutional support and the pressures of filling systemic gaps.

Community health workers (*n* = 5) were asked to reflect on their roles within the community and the types of care they provided, often informally or in semi-formal arrangements. They described challenges related to limited institutional recognition, lack of formal training, and difficulties mediating between local health beliefs and formal medical advice. Their accounts also offered critical observations about barriers patients faced in accessing care, as well as personal reflections on the realities of healthcare delivery in rural or underserved areas.

All interviews were conducted in Italian, with the use of Calabrian dialects when appropriate. They were audio-recorded with permission and transcribed verbatim. When necessary, transcripts were translated into English for analysis. Interview duration ranged from 45 min to 2 h.

3. Fieldnote documentation.

Supplementary to interviews, fieldnotes were maintained daily, providing contextual descriptions of healthcare infrastructure, environmental conditions, and community practices. These notes also captured reflexive observations by the lead ethnographer regarding positionality, rapport, and emergent themes.

### Positionality of the researchers and participants

Throughout fieldwork, the researcher maintained a reflexive orientation regarding their positionality as a healthcare-affiliated ethnographer embedded in both academic and clinical settings in Calabria. Prolonged engagement enabled the cultivation of trust and iterative interpretation. While participants were not formally involved in coding or analysis, preliminary findings were discussed informally during follow-up visits and community interactions, allowing for participant feedback on interpretations. These conversations—particularly with community health workers and recurring patient interlocutors—helped clarify meanings, confirm the salience of emerging themes, and guide the representation of illness narratives with cultural fidelity.

### Data analysis

Data were analyzed using inductive thematic analysis informed by grounded theory. Coding and initial theme development were conducted by D.C., who maintained detailed analytic memos throughout the process. These were discussed during peer debriefing sessions with R.S. and two external qualitative collaborators, ensuring triangulation and minimizing interpretive bias. R.S. contributed to theme refinement, particularly around systemic and policy-related dimensions.

The analysis proceeded through the following stages:

1. Open coding: Line-by-line coding of transcripts and fieldnotes to identify recurring patterns and concepts.
2. Axial coding: Grouping of codes into higher-order themes reflecting relationships and contrasts.
3. Selective coding: Integration of themes into a coherent explanatory framework, emphasizing the intersections of cultural belief systems, gender dynamics, and structural constraints.

Coding was performed manually by the lead researcher and verified through peer debriefing sessions with two qualitative research collaborators not involved in data collection.

### Ethical considerations

This study was conducted in accordance with the ethical standards of the Declaration of Helsinki, and approved by the Institutional Review Board of the Interuniversity Center of Phlebolymphology (CIFL) International Research and Educational Program in Clinical and Experimental Biotechnology (ER.ALL.2018.73 A.). Verbal informed consent was deemed appropriate due to the low-risk nature of the study and the potential discomfort among older participants in signing formal documents. All data were anonymized, and identifiers were removed from transcripts to ensure confidentiality.

## Results

The study sample consisted of 27 participants, predominantly women (17 out of 27), reflecting both the higher prevalence of CVD among women and their central role in caregiving and community health activities. The patients with CVD (*n* = 18) (Table 1) had an age range of 50–85 years, aligning with epidemiological data indicating increased CVD prevalence in aging populations. Among the 18 patients, 11 were women. Many of these women reported sociocultural experiences of body shame and social withdrawal. Rural participants (*n* = 17) were overrepresented among both patients and caregivers, consistent with the study’s goal of foregrounding structural disparities in underserved communities (Table 2). This rural skew also contributed to findings around care delays, traditional health beliefs, and logistical challenges accessing specialist care (Healthcare Marginality and Resourcefulness).

**Table 1:** Demographics of patients.

ID	Age	Gender	Residence	Occupation (former/current)	Notes on CVD experience
P1	68	M	Rural	Farmer	Normalized symptoms; delayed diagnosis
P2	74	F	Rural	Homemaker	Attributed symptoms to cold weather
P3	80	F	Urban	Retired seamstress	Sought help after ulcer complications
P4	72	F	Rural	Church volunteer (retired)	Social withdrawal due to leg appearance
P5	75	M	Urban	Retired bus driver	Rejected help-seeking; linked to masculinity
P6	79	F	Rural	Homemaker	Delayed care; historical silence on symptoms
P7	77	F	Urban	Retired shop assistant	Bureaucratic frustration with system delays
P8	69	F	Rural	Agricultural worker (retired)	Used traditional remedies; biomedical mistrust
P9	82	M	Rural	Farmer (retired)	Dependent on neighbor for care access
P10	64	M	Urban	Mechanic	Minimal symptom acknowledgment
P11	50	F	Urban	Secretary	Open to treatment; influenced by daughter’s support
P12	73	M	Rural	Mason	Avoided clinics; relied on folk interpretations
P13	85	F	Rural	Widowed; homemaker	Community volunteer provided wound care
P14	55	F	Urban	School janitor	Believed symptoms tied to religious punishment
P15	60	M	Urban	Shop owner	Minimal health engagement; no follow-up visits
P16	67	F	Rural	Artisan	Used herbal treatments before seeking help
P17	71	F	Urban	Caregiver (retired)	Sought early help; faced long wait times
P18	70	M	Rural	Fisherman (retired)	Helped by son; refused compression therapy

To enhance interpretive clarity, we re-examined patient profiles in Table 1 through the lens of illness trajectories and care patterns. Specifically, we identified emergent categories such as delayed diagnosis, avoidant behavior, early help-seeking, and mixed traditional-biomedical engagement. Correspondingly, care patterns were categorized into biomedical-only, traditional remedies, mixed use, or no active care. This typological framing allows for a more coherent understanding of how sociocultural beliefs, gender norms, and structural barriers shaped distinct pathways through illness. By highlighting these patterns, the table moves beyond descriptive accounts to offer an analytical overview of the diverse ways patients navigated their condition.

**Table 2:** Demographics of caregivers.

ID	Age	Gender	Relation to patient	Residence	Role in care	Challenges reported
C1	42	F	Daughter	Urban	Transport, appointment scheduling	Struggled to convince mother to seek care
C2	68	M	Spouse	Rural	Dressing wounds, home support	Mistrusted hospital care
C3	35	F	Niece	Rural	Medication management, transport	Conflict with elder’s preference for folk remedies
C4	56	F	Daughter-in-law	Urban	Language mediator, clinic visits	Felt unsupported by local services

The inclusion of community health workers (CHWs) added a systemic perspective to the patient narratives (Table 3). Notably, four out of five CHWs were women embedded in rural settings, which complemented the ethnographic emphasis on informal care infrastructures and revealed the feminization of both unpaid and semi-formal health labor in marginalized regions.

**Table 3:** Demographics of CHWs.

ID	Age	Gender	Residence	Employment status	Main duties	Reported barriers
CHW1	59	F	Rural	Volunteer	Home visits, emotional support	Lack of training and institutional support
CHW2	45	M	Urban	Semi-formal	Health education, translation	Burnout and bureaucratic constraints
CHW3	63	F	Rural	Informal network	Dressing changes, medical liaison	Unrecognized labor, financial insecurity
CHW4	42	F	Rural	Volunteer	Herbal remedy advice, prayer circles	Navigating dual logics (folk vs. medical)
CHW5	54	M	Urban	Public-sector adjunct	Monitoring appointments, transport logistics	Marginalized by formal healthcare actors

This demographic composition provided a robust foundation for understanding the intersectional dimensions of CVD in Calabria—particularly how gender, geography, and age shaped the illness experience and access to care. The demographic tables presented below (Tables 1, 2 and 3) provide a structural overview of the participant pool. These data illuminate key analytical patterns—for example, the predominance of rural patients and caregivers, which aligns with the thematic focus on infrastructural neglect, and the feminization of caregiving, which underpins the discussion on gendered labor and health responsibility.

### Ethnographic findings: a multi-level experience of chronic venous disease

The illness experience of CVD in Calabria emerged across three interrelated levels—personal, social, and systemic—each shaping how symptoms are interpreted, managed, and acted upon. Rather than a linear diagnostic trajectory, patient narratives revealed a layered, culturally embedded process of meaning-making and care navigation.

- Personal level: aging, interpretation, and spiritual meaning.

Many patients framed early symptoms—such as heaviness, itching, or cramping in the legs—not as pathological signs requiring medical attention, but as expected features of aging or fatigue. Rather than failing to notice symptoms, individuals interpreted bodily discomfort through culturally embedded frameworks that associated it with time, labor, and seasonal rhythms. In rural areas, leg pain was commonly attributed to physical work or cold weather. These interpretations led not to ignorance, but to a reframing of symptoms as non-medical and non-urgent, contributing to delayed diagnosis and formal treatment. Religious and spiritual beliefs often influenced these interpretations, casting illness as a moral or divine challenge and shaping help-seeking behaviors.

“I thought it was just my legs getting tired from working the land all day. Who goes to the doctor for tired legs?” (Patient, Man, 68, rural).

“In the village, we say when your legs swell in winter, it means the cold has entered your bones. It’s not seen as an illness.” (Patient, Woman, 74, rural).

“I didn’t go to the clinic until the wound started leaking and wouldn’t heal. That’s when I knew it was serious.” (Patient, Woman, 80, urban).

These perspectives reveal how symptom normalization—rooted in both local knowledge and spiritual worldviews—delays recognition of pathology until more severe manifestations emerge.

Caregiver perspectives echoed these themes, highlighting the challenges of convincing elders to seek formal care:

“My mother always said the leg pain was just from working hard. It was hard to get her to see the doctor before things got worse.” (Caregiver, Niece, 35, rural).

“In our family, we rely a lot on home remedies first. Only when it doesn’t improve do we think about going to the hospital.” (Caregiver, Daughter-in-law, 56, urban).

Community Health Workers described their role in bridging this gap:

“Many patients wait until it’s urgent because they see these symptoms as part of aging or punishment from God. We try to educate them, but it’s slow, and trust takes time.” (CHW, Female, 42, rural).

“People respect traditional explanations. We work with local priests and elders to explain why early treatment matters, but the beliefs run deep.” (CHW, Male, 45, urban).

These complementary accounts from caregivers and CHWs reinforce how deeply ingrained cultural interpretations and spiritual beliefs shape symptom perception and help-seeking patterns. The normalization of symptoms within aging and labor narratives is not simply individual but embedded within family and community practices, underscoring the importance of culturally sensitive health education and intervention strategies.

b. Social expectations shaped the embodied experience of CVD in gendered and generational ways.

Women often reported shame and body image concerns related to visible symptoms such as varicose veins or ulcers. These concerns led to concealment and social withdrawal, reflecting broader norms of feminine propriety and physical appearance. Men, conversely, tended to underreport symptoms or reject treatment, aligning with ideals of stoicism and masculine endurance. These gendered dynamics limited early intervention and open discussion of illness. Generational differences further complicated the social landscape: younger women were somewhat more open to discussing symptoms, though still influenced by expectations to maintain composure and beauty. Elderly women often expressed regret over delaying care due to internalized stigma, while caregivers described emotional and practical labor conducted in silence.

“I didn’t want anyone to see my legs. I stopped going to church because I couldn’t bear the looks.” (Patient, Woman, 72, rural).

“My son told me to see a doctor, but I said, ‘Men don’t cry over aching legs.’” (Patient, Man, 75, urban).

“When I was young, no one talked about these things. We just suffered and stayed quiet.” (Patient, Woman, 79, rural).

“My mother-in-law would say, ‘Don’t fuss. It’s just the veins acting up again,’ but I could see she was in pain every evening.” (Caregiver, Woman, 56, urban).

“The women I visit rarely show me their legs at first—they apologize for how they look, as if it’s their fault.” (CHW, Female, 42, rural).

“Sometimes I feel like I’m carrying her pain too, but I can’t complain—it’s expected of us.” (Caregiver, Daughter, 42, urban).

These perspectives underscore how gendered and generational norms are not only internalized by patients but also distributed across the caregiving network. The burden of shame and silence becomes a shared emotional landscape, shaping how illness is managed, disclosed, or hidden. Caregivers—especially female kin—are often caught between cultural expectations and clinical urgency, navigating their roles through subtle emotional labor and symbolic deference. Meanwhile, CHWs serve as cultural brokers, mediating between biomedical care and social propriety. Together, these narratives highlight the moral and aesthetic dimensions of chronic illness, showing that CVD is not only experienced in the body but also through the gaze of others, social scripts, and intergenerational memories of suffering.

During a rural home visit, a caregiver discreetly adjusted her mother-in-law’s compression stockings while recounting how the elder woman refused to let anyone else see her ulcers. The room was dimly lit; mirrors were covered. “She says the veins make her look cursed,” the caregiver whispered. (Fieldnote, October 2022).

These narratives illustrate the social silencing of illness and the weight of normative expectations on bodies, behaviors, and caregiving.

c. Systemic level: structural violence and resourceful navigation.

Beyond personal and social framings, the experience of CVD was shaped by systemic health inequities. Patients described profound frustrations with the healthcare system, citing long travel distances, limited access to specialists, and fragmented follow-up care—especially in rural and economically deprived areas. These structural barriers contributed to delays and discontinuities in treatment. In response, families and communities developed resourceful strategies to cope with systemic neglect. Informal caregiving networks, traditional remedies, and reliance on community health workers became essential. While these responses reflected resilience, they also exposed the uneven burden placed on marginalized communities to compensate for institutional failure.

“I waited four months to see the specialist, and when I did, he told me to come back in six. I was desperate.” (Patient, Woman, 77, urban).

“If it weren’t for my neighbor, I wouldn’t have made it to the city hospital. She’s like family now.” (Patient, Man, 82, rural).

“I use an old remedy my grandmother taught me. We don’t always trust the pills they give us.” (Patient, Woman, 69, rural).

“Sometimes we have to act as nurses, drivers, and psychologists all in one. The system leaves us no choice.” (Caregiver, Daughter, 42, urban).

“Many of us CHWs walk for hours to check on patients. We bring bandages or make calls to doctors, but we can’t replace a proper health system.” (CHW, Woman, 42, rural).

These additional perspectives highlight how systemic shortcomings extend the burden of care onto informal and often unpaid actors, including family caregivers and CHWs. Their testimonies underscore not only the resilience of local networks but also the moral and logistical exhaustion that comes from filling the gaps left by fragmented and unequal healthcare infrastructures. During a morning visit to the Catanzaro outpatient clinic, I observed a woman in her seventies arrive alone by bus, visibly fatigued and clutching a folder of worn medical documents. She stood silently at the counter for several minutes before a nurse acknowledged her, only to be redirected to another department down the corridor. She whispered, “They always say come back later,” before exiting without receiving care. (Fieldnote, March 2023).

The theme of resourcefulness can thus be understood not only as cultural adaptation, but as a survival strategy in the face of structural violence and chronic institutional failure. Community health workers—operating without stable institutional support—embodied this precarious interface between formal systems and local needs.

## Discussion

This ethnographic study of CVD in Calabria shows that illness is shaped by social, cultural, and structural factors, not just medical ones. Urban patients had better healthcare access, while rural patients faced greater barriers and often turned to traditional care. Caregiving, mainly by women, revealed tensions between cultural norms and clinical needs. The study emphasizes the need for context-sensitive health approaches and expands the focus beyond clinical management to include the lived “illness experience” according to Kleinman et al.^11^—how people interpret symptoms, seek care, and navigate health systems. A key finding is the normalization of early symptoms, often seen as natural aging or hard work rather than illness.

This culturally grounded interpretation delayed the identification of disease and engagement with care systems. Such a pattern mirrors findings in other chronic conditions where bodily discomfort is contextualized through local idioms of aging or exertion^15^. The research highlights that early symptoms of CVD, like leg heaviness or swelling, are often seen as normal aging or effects of hard work, rather than signs of illness. This cultural perspective delays disease recognition and seeking medical care, a pattern also seen in other chronic conditions.

For instance, in the Philippines, Mendoza et al.^16^ documented how hypertension was commonly viewed in naturalistic terms—understood as a normal consequence of emotional stress, aging, or lifestyle rather than as a biomedical condition requiring structured care. Most caregivers were female kin—daughters, nieces, or daughters-in-law—who often negotiated between elder resistance to biomedical care and their own attempts to ensure treatment compliance.

The tensions were especially pronounced in multigenerational households, where respect for elder autonomy sometimes clashed with the caregivers’ sense of responsibility and frustration. These dynamics resonate with broader anthropological debates on aging and care. As Buch^17^ notes, aging is not simply a biological process but a socially mediated one, where practices of care reflect and reproduce ideas about personhood, moral obligation, and value. In Calabria, caregiving was not only a functional necessity but a moral performance of familial duty, shaped by gendered expectations and local scripts of respect.

Furthermore, Corwin et al.^18^ argue that the dominant ideal of “successful aging”—which emphasizes independence, productivity, and cognitive clarity—can marginalize those whose aging is marked by decline, dependence, or non-normative trajectories. This was echoed in how older individuals in this study resisted care, not only out of mistrust or fatalism but also as an assertion of dignity within a cultural context that associates care-receiving with loss of status.

Understanding these dynamics involves viewing aging as a process of negotiating identity, autonomy, and relationships, rather than simply decline. This perspective shapes treatment-seeking behavior, often leading individuals to delay medical care in favor of traditional or self-care approaches.

In Calabria, early CVD symptoms were often seen as normal signs of physical wear, with medical care considered unnecessary unless daily function was severely affected. This perspective reflects a local understanding of health that values functional ability over clinical diagnosis. As a result, simply providing medical information is insufficient—health interventions must also address these cultural beliefs about when a body is considered truly ill^19,20^.

The gendered dimension of CVD emerged as another critical theme. For women, visible symptoms such as varicose veins or ulcers triggered shame and social withdrawal, particularly in public and religious spaces. This aligns with literature on chronic illness and embodiment, which argues that visible bodily signs often become moralized, particularly for women^21^. In patriarchal contexts, the female body remains a site of social scrutiny, and any deviation from aesthetic norms—such as blemished or discolored skin—can result in stigma^22^. Men, conversely, adhered to ideals of stoicism and endurance, often dismissing symptoms and avoiding healthcare engagement. This behavior reflects hegemonic masculinity norms that equate vulnerability with weakness, as documented in other ethnographies of men’s health^23^. Importantly, these gendered scripts do not simply influence individual behavior but shape entire diagnostic trajectories, as both men and women delay care for reasons deeply tied to societal expectations^24^.

The study reveals that gender plays a key role in how people experience and respond to CVD. Women often internalized symptoms with shame and withdrew socially due to cultural ideals of beauty and respectability, while men tended to deny or downplay symptoms to uphold norms of strength and independence. Despite these differing expressions—shame versus stoicism—both led to delays in seeking care and worsening health. This highlights how social and cultural expectations around gender shape illness experiences and discourage timely medical intervention.

Healthcare providers should move beyond gender-neutral approaches and consider how cultural ideas of masculinity and femininity influence how patients recognize and express symptoms^25^. The study also highlights patients’ deep sense of marginalization, as many reported facing bureaucratic hurdles, long wait times, and logistical barriers like distance and lack of transportation. These experiences are emblematic of what Paul Farmer^10^ described as “structural violence”—a form of harm embedded in social structures that systematically disadvantage certain populations.

Structural inequalities emerged as a central barrier to care, with patients frequently describing long wait times, confusing bureaucratic procedures, and inadequate transportation—particularly in rural areas. These challenges were not isolated inconveniences but forms of structural violence that delayed treatment and deepened suffering, especially for economically insecure older adults. Ethnographic accounts—such as an elderly woman turned away after traveling hours for an appointment, or a man reliant on neighbors for transport—illustrate how systemic neglect discourages help-seeking. These findings align with Paul Farmer’s concept of structural violence^10^ and Veena Das’s work^13^ on bureaucratic indifference, showing how institutional inefficiencies and infrastructural gaps compound vulnerability and normalize untreated illness in marginalized regions.

In Calabria, the chronicity of CVD is not only a function of venous dysfunction but also of systemic neglect. A woman waiting four months for a specialist appointment is not simply enduring illness; she is subject to an ongoing structural failure that prolongs suffering and deepens social exclusion^26^. Chronic illness, in this sense, becomes a metaphor for the region’s political and economic marginalization—a slow, unrelenting reminder of inequality^27^. Healthcare reform in such contexts must go beyond improving clinical guidelines or increasing clinic numbers. It must address the foundational issues of accessibility, infrastructure, and equity^28^. Investing in mobile health units, telemedicine tailored to older adults, and incentivizing rural medical practice are crucial steps toward reducing structural gaps.

Despite the limitations of formal healthcare, patients in Calabria exhibited remarkable resilience through informal caregiving networks. Neighbors, family members, and community health workers played indispensable roles in facilitating care^29^. This echoes findings from other marginalized communities, where informal caregiving serves as a compensatory mechanism for systemic gaps^30^. Importantly, the role of CHWs was both practical and symbolic. They bridged the gap between medical institutions and local knowledge systems, offering not just logistical support but emotional validation.

However, CHWs often operated without formal recognition, training, or compensation—conditions that undermine their sustainability and legitimacy^31^. Policy must formally integrate CHWs into the healthcare system, providing training and financial support while preserving their community-embedded ethos. This integration would not only enhance patient outcomes but also promote trust in healthcare systems that have long been perceived as distant and unresponsive^32^. The coexistence of biomedical and traditional health beliefs adds another layer of complexity to CVD management^33^. While some participants trusted pills and compression therapy, others preferred herbal poultices, faith healing, or religious pilgrimage.

These pluralistic strategies are not irrational but reflect an adaptive logic rooted in history, accessibility, and lived experience. Rather than dismissing non-biomedical practices, public health efforts should engage them constructively. Community health interventions that incorporate local remedies—after vetting for safety—could improve compliance and cultural resonance^34^. Furthermore, working alongside local religious leaders or traditional healers could foster community buy-in for preventive programs, particularly in areas where biomedical mistrust is high. In multigenerational households, caregiving became a site of negotiation between younger and older family members. Younger caregivers often encouraged biomedical treatment, while elders clung to traditional beliefs or displayed fatalism^35^.

These tensions reflect not just generational divides but differing health literacies, access to information, and degrees of system trust. This dynamic provides a critical insight for public health planning: interventions should not target individuals in isolation but consider the family as a unit of care. Educational materials, home visits, and care coordination should be designed with intergenerational dialogue in mind. Programs that empower younger caregivers while respecting elder autonomy may prove more effective than one-size-fits-all outreach^36^. The findings underscore a crucial ethical imperative: healthcare systems must treat patients not just as clinical cases but as individuals situated within complex cultural and social worlds.

Cultural competence, often treated as a secondary consideration in medical training, should be central to chronic disease management^37^. Clinicians must learn to ask not only “What are your symptoms?” but also “How do you make sense of these symptoms?” Engaging with patients’ worldviews—including their spiritual beliefs, economic constraints, and gender roles—can foster more effective and compassionate care. Incorporating anthropologists or ethnographically trained professionals into healthcare teams could facilitate this shift, acting as mediators between biomedical and local epistemologies^38^. A striking insight from this research is the normalization of early CVD symptoms. Rather than viewing heaviness or swelling in the legs as pathological, many participants framed them as ordinary signs of aging or results of hard work.

While cultural competence remains important in chronic disease management, scholars like Jonathan Metzl and Helena Hansen^39^, Seth Holmes^40^, and Rebecca Hester^41^ advocate for a shift toward structural competency—an approach that equips healthcare providers to address the broader social, political, and economic forces driving health disparities. They caution that relying solely on cultural explanations can oversimplify inequalities and risk pathologizing marginalized groups. In Calabria, where issues like rural underinvestment, bureaucratic exclusion, and caregiver burden are pronounced, effective care requires more than cultural sensitivity—it calls for provider awareness of structural influences on illness. Integrating structural competency into medical education would support a more justice-oriented and equitable healthcare model.

This culturally grounded interpretation delayed the identification of disease and engagement with care systems. Such a pattern mirrors findings in other chronic conditions where bodily discomfort is contextualized through local idioms of aging or exertion. For instance, in the Philippines, Mendoza et al.^16^ documented how hypertension was commonly viewed in naturalistic terms—understood as a normal consequence of emotional stress, aging, or lifestyle rather than as a biomedical condition requiring structured care. These interpretations influenced how and when individuals sought treatment, often delaying biomedical engagement in favor of traditional or self-care practices.

These multiple understandings of CVD resonate with Annemarie Mol’s^42^ concept of the body multiple, which argues that disease is not a single, unified object but one enacted differently depending on the context—clinical, social, or experiential. In Calabria, the CVD body materialized in at least two enactments: the clinical body, identified via diagnostic criteria such as venous reflux or ulceration; and the experiential body, characterized by sensations like heaviness, fatigue, or shame, which were often interpreted through idioms of labor, gender, or aging. As Lasco et al.^43^ illustrate in their application of Mol’s^42^ framework to hypertension, such multiplicity shapes therapeutic itineraries and the thresholds at which individuals deem care necessary. Similarly, Calabrian patients did not passively delay care but actively participated in a culturally situated enactment of illness—one that may not align with clinical urgency but makes sense within their lived realities.

The normalization of early CVD symptoms reflects both cultural beliefs and practical responses to structural barriers. Many participants minimized symptoms to avoid the high costs, long travel, and inefficiencies of the healthcare system, especially in rural areas. This coping strategy helped preserve autonomy and livelihoods. Although urban patients had better access, they still faced bureaucratic delays. In both settings, care was often delayed, resulting in prolonged suffering and under-treatment.

The extended six-year fieldwork undertaken in this study is methodologically justified for several interrelated reasons. First, CVD is a slow-progressing condition that unfolds over many years, often beginning with normalized or stigmatized symptoms that evolve gradually into more serious complications. Ethnographically capturing this progression required longitudinal engagement aligned with the temporal scale of the disease, as similarly reflected in the 13.4-year follow-up of the Edinburgh Vein Study^44^. Second, the field site in Calabria posed considerable contextual challenges, including systemic healthcare inequities, rural isolation, and enduring traditional health beliefs, all of which necessitated prolonged, iterative rapport-building—particularly among older and marginalized participants. Third, there is precedent for such long-term ethnographic engagement in comparable research contexts, where scholars have investigated chronic illness, medical mistrust, and social precarity over extended periods^45–47^. Finally, this study represents the first ethnographic investigation of CVD, requiring time not only to establish trust but also to develop original conceptual frameworks bridging biomedical and sociocultural perspectives. Taken together, these factors affirm that multi-year fieldwork was essential to generating the depth, validity, and ethical rigor necessary to address the complex realities surrounding chronic illness in underserved contexts.

### Limitations and directions for future research

While this study provides rich insights, it also has limitations. The ethnographic scope, though deep, is geographically restricted to two Calabrian communities. Future research could conduct comparative studies across other regions in Southern Europe or extend to immigrant populations in urban centers, exploring how experiences of CVD differ across cultural and political landscapes. Moreover, longitudinal ethnographies could illuminate how patients’ perceptions and behaviors evolve over time—especially in response to policy changes, new medical technologies, or shifting cultural norms. The inclusion of healthcare professionals as ethnographic subjects could also enrich understanding of systemic challenges and provider-patient dynamics.

## Conclusion

Ethnography provides critical insight into the lived dimensions of Chronic Venous Disease. In Calabria, sociocultural determinants—from aging narratives to structural inequalities—profoundly shape patient trajectories. Future research and health interventions must move beyond the clinic to fully engage with the social worlds in which disease unfolds. By recognizing the human stories behind chronic illness, we can design more inclusive, equitable, and effective healthcare systems.

## Data Availability

Data is provided within the manuscript.
